# Osteolytic bone lesions, severe hypercalcemia without circulating blasts: unusual presentation of childhood acute lymphoblastic leukemia

**DOI:** 10.11604/pamj.2017.26.244.10506

**Published:** 2017-04-28

**Authors:** Achour Bechir, Regaieg Haifa, Ben Abdelkader Atef, Bouslema Emna, Achour Asma, Ben Sayed Nesrine, Ben Youssef Yosra, Khelif Abdrrahim

**Affiliations:** 1 Department of Hematology, Farhat Hached Hospital, Sousse Tunisia; 2 Department of Anatomopathology, Farhat Hached Hospital, Sousse Tunisia; 3 Department of Radiology, Fattouma Bourguiba Hospital, Monastir Tunisia

**Keywords:** Acute lymphoblastic leukemia, hypercalcemia, childhood, osteolytic bone lesions

## Abstract

Hypercalcemia and severe osteolytic lesions are rare complications of acute lymphoblastic leukemia (ALL) in childhood. We report a case of a 3 years old boy who presented with prolonged fever, nausea, vomiting and increasing lower limbs pain. Skeletal X-rays and CT scan showed severe osteolytic lesions of the skull and extremities. Her physical examination showed multiple cervical lymph nodes. In laboratory tests, he had severe hypercalcemia. Parathyroid hormone (PTH) was not elevated. Despite the absence of circulating blasts, bone marrow biopsy revealed B-precursor (ALL). Hypercalcemia was initially treated with intravenous isotonic sodium chloride solution and diuretics but the serum calcium level normalized only after the beginning of corticosteroids and chemotherapy. The child responded initially to chemotherapy and eventually relapsed and died of septic shock. Acute leukemia must be considered in differential diagnosis in patients with hypercalcemia. A detailed examination even when there no circulating blasts in their peripheral blood smear, and if in doubt bone marrow aspiration should must be taken into consideration.

## Introduction

Acute lymphoblastic leukemia (ALL) is the most common form of acute leukemia in children [1]. It usually results in signs of bone marrow failure or tumor syndrome [2]. Hypercalcemia is a rare metabolic complication of pediatric ALL [1]. Here we report a case of ALL with severe hypercalcemia, disseminated osteolytic lesion, and absence of circulating blasts.

## Patient and observation

**Figure 1:**
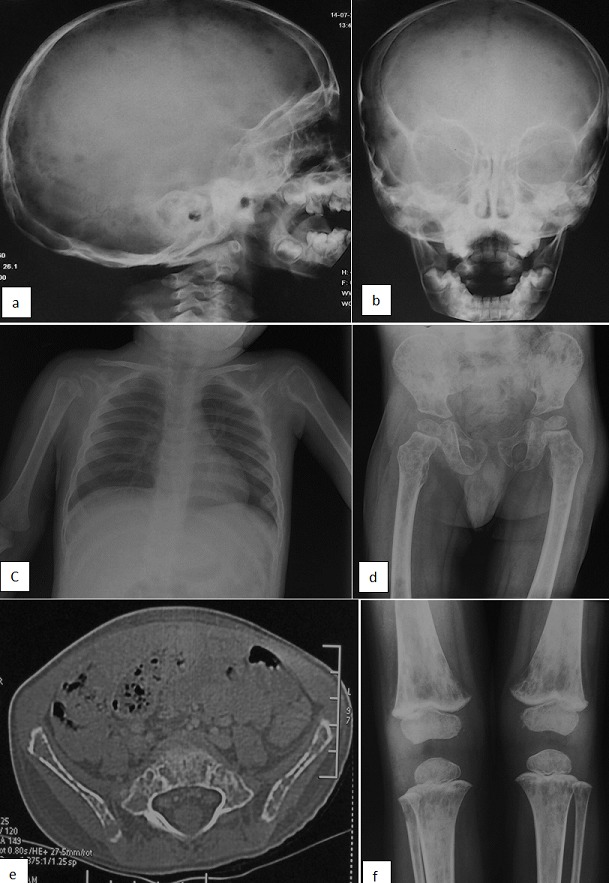
(A,B) multiple osteolytic lesions in the skull, humerus, pelvic bone, both femurs, and both tibias; Skull X-ray (front and profile): reveals multiple lytic lesions; (C) front chest X-ray revealed proximal metaphyseal humoral lytic lesion responsible for fracture; (D) X-ray reveals multiple osteolytic lesions in pelvic bone and both femurs; (E) lumbosacral and pelvis CT Scan revealed diffuse lytic lesions and permeative bony lesions in iliac bones; (F) X-ray of knee joints reveals metaphyseal lytic lesions in both distal femurs and proximal tibiae

**Figure 2:**
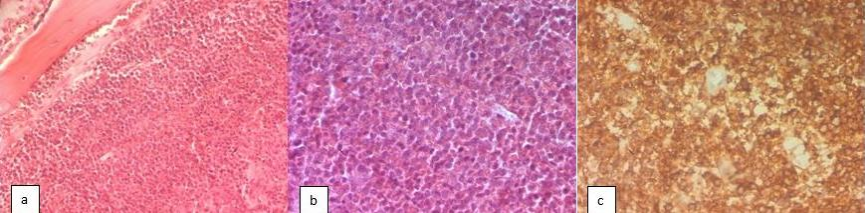
Acute lymphoblastic leukemia-histology; (A) HE stain x 50; diffuse infiltration of the bone marrow by small round cells; (B) HE stain x 100; nuclei are convoluted with fine chromatin and frequent mitosis; (C) CD10 stain x 100; diffuse expression in neoplastic cells

Acute lymphoblastic leukemia (ALL) is the most common form of acute leukemia in children [1]. It usually results in signs of bone marrow failure or tumor syndrome [2]. Hypercalcemia is a rare metabolic complication of pediatric ALL [1]. Here we report a case of ALL with severe hypercalcemia, disseminated osteolytic lesion, and absence of circulating blasts. Case report A 3 years old boy was admitted to our department because of prolonged fever, nausea, vomiting and increasing pain in both lower limbs. On physical examination, he appeared pale, apathetic, with infracentimetric cervical lymphadenopathy and no cardiovascular symptoms. His lower limbs appeared painful without any signs of inflammation. Initial laboratory investigations revealed white blood cells count of 6400/ml (with neutrophil 3500/ml, lymphocytes 2400/ml), haemoglobin level of 5.9 gr/dl and platelets count of 164,000/ml. Peripheral blood smear showed no blasts or abnormal cells. The erythrocyte sedimentation rate was high (33 mm), the C-reactive protein level was high (65 mg/ml). Serum calcium was 4.65 mmol / l, with normal serum creatinine, liver function tests phosphorus, albumin 27.8g/l and serum LDH. Serum parathormone was low (2.08 ug/ml). Unfortunately we didn't have 25(OH)-vitamin D, calcitonin and PTH-related protein (PTHrP) levels. The thyroid function were high for throid-stimulating hormone (13 mIU/l) and normal for FT4 (14mg/ml). Urine calcium to creatinine ratio was 4.32 and urine phosphorus was 2.08 mmol/l. Abdominal ultrasound was normal. Skeletal radiographs and computed tomography (CT) scan of the skull, thorax and abdomen revealed osteolytic lesions in the skull, pelvis and long bones Figure 1. Despite the absence of circulating blasts, bone marrow aspiration and biopsy studies led to the diagnosis of B-cell precursor ALL (Diffuse CD10 positive, focal CD20 positive, CD79a negative, CD34 negative and TdT negative) Figure 2. Cerebrospinal fluid (CSF) cytology was negative for malignant cells. Cytogenetics by conventional karyotyping showed a deletion of 12p (del(12) (p13) [7]/46,XY [13]). The hypercalcemia was initially treated with intravenous isotonic saline and diuretics, but the serum calcium level normalized only after the beginning of specific chemotherapy according to the EORTC protocol. The patient was in remission for one year and had a good clinical condition and finally relapsed and died of septic shock.

## Discussion

Hypercalcemia is a rare metabolic complication and occurs in less than 1% of children with cancer [3]. In the childhood, vitamin D intoxication, primary hyperparathyrodism, immobilization and malignancy are the main causes of hypercalcemia [3]. The incidence of hypercalcemia associated with acute leukemia of children ranging between 0.6 and 4.8% of cases [4, 5]. Two general mechanisms explain malignancy associated hypercalcemia: osteolytic lesions due to direct invasion of the skeleton by tumor cells and ectopic production of circulating factors that activate osteoclastic bone resorption. The main factors is the osteolytic PTH-related protein (PTHrP) [3]. ALL presenting with hypercalcaemia has a distinctive clinical profile, and most of the reported cases show certain common characteristics [6-8]. It is observed in most children concerned similar characteristics such as older age (10-20 years), normal or low white blood cell (WBC) counts with rare or absent circulating blasts on peripheral blood smear. The blasts characteristically show a pre-B ALL phenotype with aberrant expression of CD13 and other myeloid antigens and often are positive for t(17;19) on cytogenetic studies [6-8]. In our observation all these characteristics are present apart from age, our patient was young and del(12) (p13) on cytogenetic studies. Another common accompanying finding in ALL patients presenting with hypercalcemia is disseminated osteolytic lesions [8]. In our patient, osteolytic lesions were found interestingly multiple osteolytic lesions were seen in the skull, humerus, pelvic bone, both femurs, and both tibias.

Our case as well as the review of the literature confirms that in childhood ALL presenting with hypercalcemia, the main symptoms are non-specific: anorexia, vomiting, abdominal pain, constipation, confusion, bradycardia and these signs may precede those related to intramedullary invasion by blast cells [2]. When hypercalcemia is confirmed, urgent interventions to prevent severe renal, pancreatic, or cardiac complication are necessary [9]. The treatment of hypercalcemia is based on two principles: lowering the serum calcium concentration and treating the underlying disease [2]. The symptomatic treatment combines intensive hydration and diuretics. Calcitonin is also effective because it inhibits bone resorption and increases the excretion of urinary calcium. Calcitonin acts rapidly but her effect is short-lived and patients develop tolerance due to down regulation of calcitonin receptors on osteoclasts. If this treatment fails, corticosteroids may be useful. It have an important role to not only reduce hypercalcaemia but also have an antileukaemic effect [10]. Bisphosphonates are being used more frequently in adults with malignant hypercalcemia. There are few studies about the use of bisphosphonates in the treatment of childhood hypercalcemia [11]. These agents decrease serum calcium by inhibiting osteoclast-mediated resorption of bone. The effect of bisphosphonates takes about 16- 48 hours to commence and serum calcium levels usually normalize within 3-8 days [9]. Our patient didn't respond initially to intense intravenous isotonic sodium chloride solution and furosemide. Good control of the hypercalcemia was obtained after initiation of corticosteroids and antileukemic treatment. In our patient, we didn't administer bisphosphonates. He remained in remission for one year, and finally relapsed and died of septic shock.

## Conclusion

The hypercalcemia is a metabolic emergency in children and it may be complicate acute leukemia at diagnosis. Therefore, a bone marrow examination should not be delayed in these cases even if the physical examination is normal and there are no circulating blasts in the peripheral smear. Once identified it can be rapidly treated with a combination of intravenous isotonic saline, diuretics, calcitonin and bisphosphonates. Early treatment of leukemia helps to correct quickly hypercalcemia along with other therapies.
